# Endobronchial valve positioning for alveolar-pleural fistula following ICU management complicating COVID-19 pneumonia

**DOI:** 10.1186/s12890-021-01653-w

**Published:** 2021-09-27

**Authors:** Pierluigi Donatelli, Fabiana Trentacosti, Maria Rosaria Pellegrino, Roberto Tonelli, Giulia Bruzzi, Alessandro Andreani, Gaia Francesca Cappiello, Dario Andrisani, Filippo Gozzi, Cristina Mussini, Stefano Busani, Gilda Valentina Cavaliere, Massimo Girardis, Elisabetta Bertellini, Enrico Clini, Alessandro Marchioni

**Affiliations:** 1 University Hospital of Modena, Respiratory Diseases Unit, Department of Medical and Surgical Sciences, University of Modena Reggio Emilia, Modena, Italy; 2 Clinical and Experimental Medicine PhD Program, University of Modena Reggio Emilia, Via Università 4, 41121 Modena, Italy; 3 University Hospital of Modena, Infectious Diseases Unit, University of Modena Reggio Emilia, Modena, Italy; 4 University Hospital of Modena, Anesthesiology Unit, University of Modena Reggio Emilia, Modena, Italy; 5 Laboratory of Experimental Pneumology, Modena, Italy; 6 Respiratory Diseases Unit and Center for Rare Lung Disease, Department of Surgical and Medical Sciences, University Hospital of Modena, Via del Pozzo, 71, 41125 Modena, Italy

**Keywords:** COVID-19, Alveolar-pleural fistula, Endobronchial valve, Pneumothorax, Klebsiella pneumoniae

## Abstract

**Background:**

The main clinical consequences of severe acute respiratory syndrome Coronavirus 2 (SARS-CoV-2) infection are pneumonia and respiratory failure even requiring mechanical ventilation. In this context, the lung parenchyma is highly prone to ventilator-related injury, with pneumothorax and persistent air leak as the most serious adverse events. So far, endobronchial valve (EBV) positioning has proved efficacious in treating air leaks with a high success rate.

**Case presentation:**

We report, for the first time, two cases of patients affected by SARS-CoV-2-related pneumonia complicated with bacterial super-infection, experiencing pneumothorax and persistent air leaks after invasive mechanical ventilation. Despite the severity of respiratory failure both patients underwent rigid interventional bronchoscopy and were successfully treated through EBV positioning.

**Conclusions:**

Persistent air leaks may result from lung tissue damage due to a complex interaction between inflammation and ventilator-related injury (VILI), especially in the advanced stages of ARDS. EBV positioning seems to be a feasible and effective minimally invasive therapeutic option for treating this subset of patients.

## Background

A case clusters of pneumonia caused by the novel severe acute respiratory syndrome coronavirus 2 (SARS-CoV-2) was first reported in Wuhan city of China in December 2019, then spread worldwide [1]. Clinical presentations of coronavirus related disease (COVID-19) vary from asymptomatic to mild influenza-like symptoms to life-threatening pneumonia, whilst multiorgan failure can also occur [1, 2]. COVID-19 acute lung injury results from exaggerated inflammation started by viral replication and then sustained by a cytokine storm that is associated with disease severity [3, 4]. Although the physiopathological features of respiratory failure caused by SARS-CoV-2 infection may be heterogeneous, in most severe cases of COVID-19, acute respiratory distress syndrome (ARDS) occurs [5]. In these patients, high elastance and severe lung inhomogeneity make the parenchyma prone to ventilator-induced lung injury (VILI) when mechanical ventilation (MV) is applied [6, 7]. Positive pressure ventilation, especially at the interface between structures with different extensibilities, may lead to harmful transpulmonary pressure causing the rupture of extracellular polymers, resulting in “barotrauma”, of which the most common form is pneumothorax [9, 10]. Despite the onset of pneumothorax requiring chest drainage having been reported in 5.9% of COVID-19 patients undergoing MV [11], only a few cases of persistent air leak (PAL) have been described to date. PAL is defined as an air leak that persists for more than 5–7 days [12]. A bronchopleural fistula (BPF) occurs when PALs are supported by a communication between the bronchus and the pleural space, whereas an alveolar-pleural fistula (APF) follows a distal origin of PALs [12, 13]. PALs are known to be the one of common complications after lung surgery [14]; however, APF is not frequently associated with an excessive stress/strain applied to the lung parenchyma by MV. Nonetheless, this clinical condition has serious consequences for patient management and outcome resulting in prolonged chest tube maintenance and hospital stay, and in a higher rate of infectious complications due to loss of sterility in the pleural space [12, 13]. Retrospective studies in patients with PALs have shown mortality rates ranging from 16 to 72% [11]. Treatment options include conservative management, surgical repair, and minimally invasive interventional pulmonology or bronchoscopic management [13, 15].

We present two cases of severe COVID-19 pneumonia complicated by bacterial pneumonia and APF secondary to VILI and treated through EBV positioning.

## Case presentation

### Case 1

**Fig. 1:**
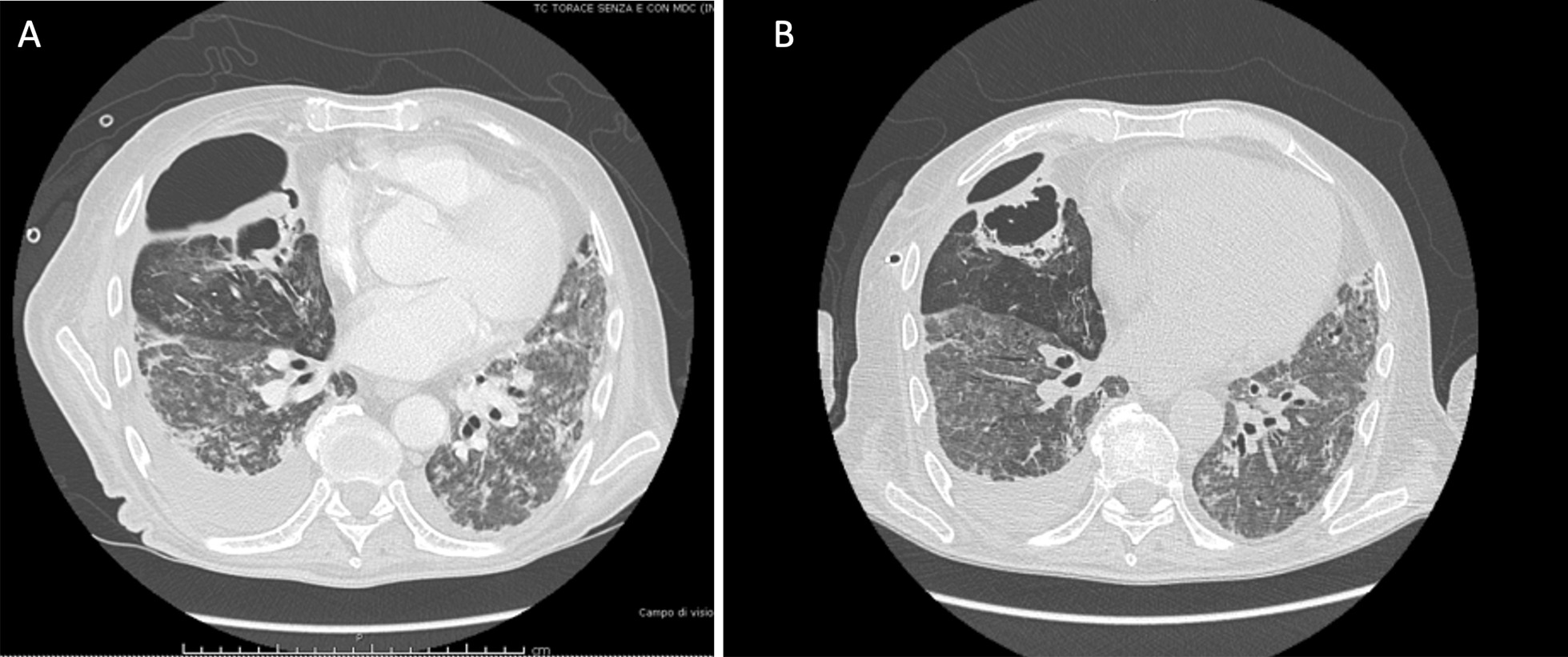
High resolution computed tomography scan in Case 1 showing right pneumothorax secondary to alveolar-pleural fistula (APF) before (panel **A**) and after (panel **B**) endobronchial valve (EBV) positioning with lung parenchyma re-expansion

In March 2020, a 67-year-old Caucasian male was admitted to the respiratory ward at the Hospital of Piacenza (Italy) presenting with fever (38 °C) and respiratory failure. His past medical history was characterized by well controlled asthma and hypertension. SARS-CoV-2 infection was confirmed by real time polymerase chain reaction (RT–PCR) assay on nasopharyngeal and throat specimens. Blood tests showed increased D-Dimer (657 ng/mL), elevated C reactive protein (CRP) (20.3 mg/dL) and procalcitonin (PCT) (0.9 ng/L). Oxygen supply was started in addition to hydroxychloroquine, azithromycin, darunavir-cobicistat, and ceftazidime. Given the rapid worsening of respiratory failure, the patient was transferred to the University Hospital of Modena where continuous positive airway pressure through a helmet was started, and Tocilizumab was infused. Following further deterioration, the patient underwent endotracheal intubation and was then transferred to the Intensive Care Unit (ICU) where invasive MV in volume-controlled mode with protective strategy was initiated. Successful extubation occurred 8 days later, thus cycles of intermittent high flow nasal cannula (HFNC) and non-invasive ventilation (NIV) were set. Due to septic shock of presumable pulmonary origin associated with chest X-ray worsening and *Klebsiella pneumoniae* bacteremia, the patient again underwent endotracheal intubation 14 days later. After 5 days, the MV regimen was complicated by the onset of right tension hydro-pneumothorax requiring decompression and insertion of chest drainage. Chest computed tomography confirmed the occurrence of a large right pneumothorax with associated pleural effusion (Fig. 1, Panel A). *Klebsiella pneumoniae* and *Enterococcus faecalis* were isolated from bronchoalveolar lavage, thus antibiotic therapy with meropenem and ampicillin was started. After 8 days, the patient was successfully weaned from MV, although the pneumothorax had not resolved with significant PAL over 3 weeks. Since an APF was assumed to be the cause, the patient was transferred to the Respiratory Unit, flexible bronchoscopy was performed, and the middle lobe was identified as the source of the air leak using a balloon occlusion test. Two valves (EBVs Zephir, size 5 mm; Zephyr® Endobronchial Valve System Pulmonx Corporation, Redwood City, CA 94063 USA) were successfully inserted into the segmentary bronchi of the middle lobe (Fig. 1, Panel B) resulting in reduction in air leak after 3 days. No residual air leak was detected in the following week, so that, once cleansing of pleural fluid was observed, drainage tube was removed successfully, as further confirmed by the chest X-ray check. In June the patient was transferred to the Hospital of Piacenza given the clinical stability, and was then discharged a month later, after pulmonary rehabilitation. A chest CT scan was performed two months later showing improvement of pneumothorax, thus suggesting APF reduction. Valves were removed 8 months later and follow-up was started. To date no recurrence of pneumothorax was reported.

### Case 2

**Fig. 2:**
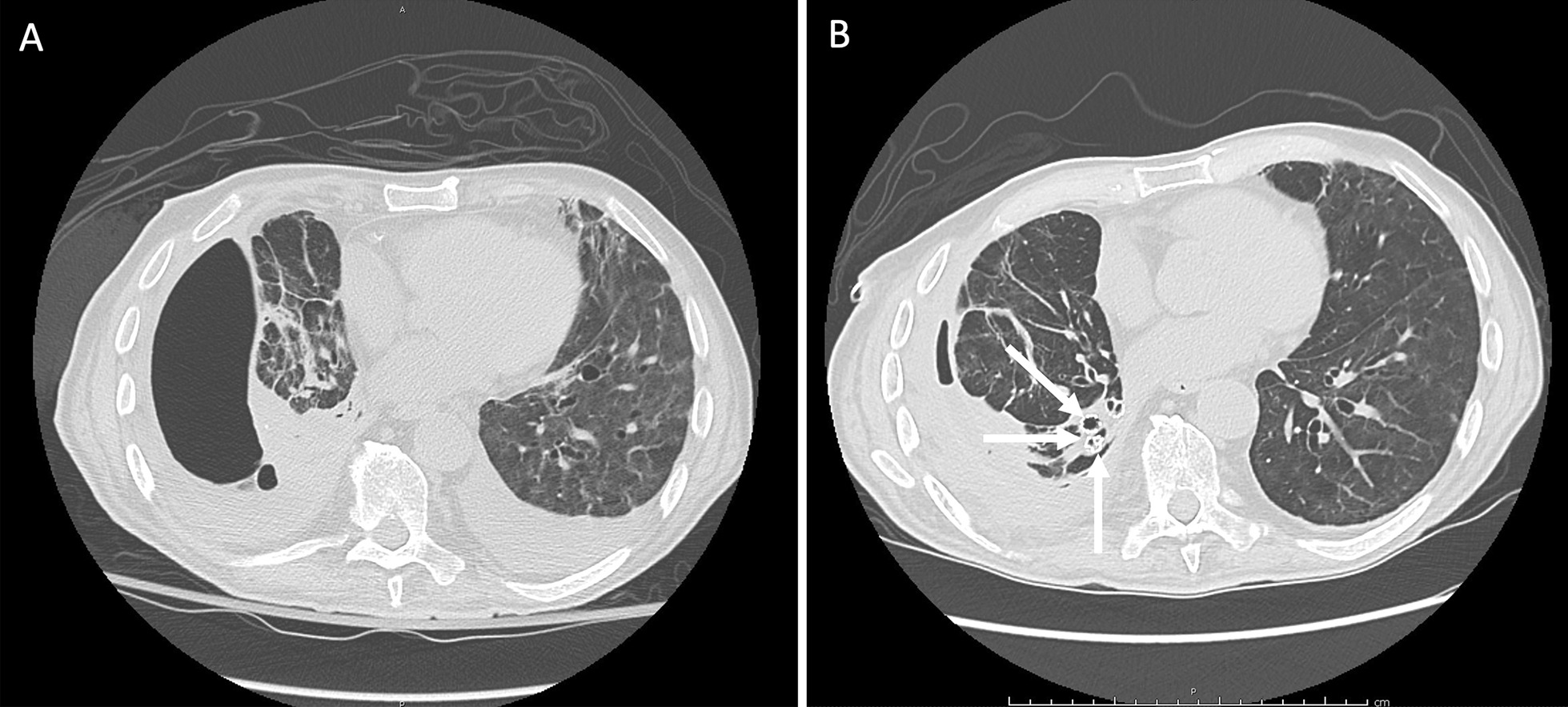
High resolution computed tomography scan in Case 2 showing right pneumothorax secondary to alveolar-pleural fistula (APF) before (panel **A**) and after (panel **B**) endobronchial valve (EBV) positioning with lung parenchyma re-expansion. Valves can be observed in the basal pyramid bronchi (withe arrows)

In March 2020, a 73-year-old Caucasian male was admitted to the Infectious Diseases Unit of the University Hospital of Modena (Italy) with persistent fever (38.8 °C) and severe acute hypoxic respiratory failure (PaO_2_/FiO_2_ = 150 mmHg). A nasopharyngeal swab (RT-PCR) tested positive for SARS-CoV-2. Chest X-ray showed bilateral interstitial infiltrates while blood tests reported increased CRP (20.8 mg/dL) and PCT (2.8 ng/mL). Oxygen supply was started along with hydroxychloroquine, azithromycin, and ceftriaxone. On the following day, the patient presented a significant worsening of gas exchange, thus continuous positive airway pressure through a helmet was started and Tocilizumab was infused. In spite of this and because of further worsening of his condition, the patient was admitted to the ICU where invasive volume-preset MV was initiated with protective strategy. A tracheostomy was performed on day 10 after ICU admission. On day 15, the occurrence of right hydro-pneumothorax (Fig. 2, panel A) required immediate insertion of a chest drainage tube. *Klebsiella pneumoniae* and *Enterococcus faecalis* were isolated from pleural fluid and successfully treated with meropenem and ampicillin. After 1 month, his clinical condition had improved markedly and weaning from MV was achieved, despite the persistence of right pneumothorax and air leak due to suspected APF. Thus, the patient was transferred to the respiratory ICU where flexible bronchoscopy was performed, and a balloon occlusion test identified the presence of APF in the right lower lobe. Three valves (EBVs Zephir, size 5 mm) were inserted into the segmental bronchi of the right lower lobe (Fig. 2, panel B). A gradual reduction of air leak was recorded over the following week, although resolution was only obtained after the further placement of a valve in the apical segment of the right lower lobar bronchus. Finally, the drainage tube was removed, and the patient was discharged on July 2020. In October 2020 he underwent endoscopic removal of endobronchial valves without any complication.

## Discussion and conclusion

COVID-19 exhibits a broad spectrum of clinical conditions, including pneumonia, ARDS, pulmonary embolism, and hearth failure, leading to the development of respiratory failure often requiring ICU management and MV (in about 20% of cases) [16]. The pathogenesis of respiratory failure is complex and covers different clinical scenarios ranging from patients with relatively spared lung compliance to patients with ARDS mechanical features. [5, 17]. Most patients admitted to the ICU with severe COVID-19 pneumonia fulfill ARDS criteria and require MV, which in turn may lead to a VILI [18]. The high stress level generated during MV associated with lung tissue inflammation can cause bronchial or alveolar ruptures followed by pneumothorax or pneumomediastinum. Gammon et al. have shown that ARDS per se is an independent risk factor, although the less aggressive use of pressure, in the lung protective strategy era, was associated with a markedly lower incidence of pneumothorax [8]. Both cases underwent MV with protective strategy (expiratory tidal volume [Vte] < 6 ml/kg of predicted body weight, plateau pressure [P_plat_] < 28 cmH_2_O, PEEP titration according to FiO_2_ schedule) and were not subjected to recruitment maneuvers. In this scenario, it may be speculated that regional factors associated to lung inhomogeneity due to advanced stages of COVID-19 related ARDS and inflammatory related tissue frailty, could have played a major role in exacerbating barotrauma, resulting in APF. In the previous SARS outbreak, barotrauma was a common complication during MV. Indeed, studies in mechanically ventilated SARS patients showed a high incidence of pneumothorax (20–30%) and pneumomediastinum (12%) [19–22]. Patients with a lower PaO_2_/FiO_2_ ratio, higher PaCO_2_, and higher respiratory rate during ICU stay were at a greater risk of developing pneumothorax [23]. The incidence of SARS-CoV-2-related pneumothorax appears to be significantly lower (5.9%) than reported in patients with SARS-CoV-1, but it can lead to a mortality of 30% [24]. Moreover, studies performed in non-SARS-CoV-2 patients with ARDS showed that overinflation and distortion of lung structures due to MV could result in emphysema-like lesions, lung cysts, and bronchiectasis [25]. In the late stages of ARDS, these lesions prevail in nondependent and caudal lung regions as confirmed by chest CT scan [26]. Furthermore, experimental studies in animals have demonstrated that MV set at high tidal volume or high PEEP and recruitment maneuvers results in air space enlargement after 2 days [27, 28].

Radiologic studies in COVID-19 with ARDS have shown that patients can develop cystic lung parenchyma changes and bullae formation during viral infection [29]. These parenchymal changes may predispose patients undergoing MV to develop pneumothorax and PALs, especially in the advanced stage when the lung has possibly undergone fibrotic changes. Since the fibrotic lung is particularly subject to static strain, the hyperinflation achieved with the addition of PEEP can facilitate enlargement of the air spaces in non-dependent areas of the lung [30]. Moreover, both cases showed the isolation of *Klebsiella pneumoniae* and *Enterococcus faecalis* from bronchoalveolar lavage (Case 1) and pleural fluid (Case 2). A bacterial super-infection may have enhanced the inflammatory mechanism of lung injury already triggered by SARS-CoV-2 infection, thus increasing the susceptibility to PALs development. Indeed, necrotizing infections of the lung caused by gram negative bacteria (namely *Klebsiella pneumoniae*) have been widely reported [31]. Given the lack of evident signs of fistulisation at bronchoscopic examination we concluded for APF diagnosis for both cases.

Traditional approaches to PALs range from watchful waiting with prolonged chest tube placement to more invasive measures including surgery. There is a recent report of two COVID-19 patients with PALs successfully treated with thoracoscopy, blebs resection, and pleurectomy [32]. Different less invasive options have been tested, such as autologous blood pleurodesis, Heimlich valve positioning, and albumin-glutaraldehyde tissue adhesives [13]. Recently a BPF related PAL was successfully treated with EBV placement [33]. To our knowledge, these are the first two cases documenting the effective use of EBVs to treat PALs due o APF in patients infected with novel SARS-CoV-2 who required invasive MV.

EBV was first administered for lung volume reduction in patients with severe emphysema [34], and it is currently the most actively performed procedure for PALs, when surgical options are not viable [13, 15]. EBV has been approved by the Food and Drug Administration to treat PALs under humanitarian use regulations, reducing airflow across the fistula and allowing healing and resolution [35]. In a multicenter study with 40 patients, Travaline et al. showed that Zephyr EBV is an effective minimally invasive device for completely treating PALs in around half of the treated patients, and complications such as valve malposition and/or expectoration, and pneumonia are quite rare [36].

Localization of PALs is challenging, and the balloon occlusion method is currently the most used approach [13]. Our two patients experienced continuous bubbling during inspiration/expiration, i.e. grade 4 according to the Cerfolio classification of air leaks [13]. After the EBV procedure, both cases showed complete resolution of PALs from APF without any need for surgery.

Although, our reported experience in COVID-19 patients with iatrogenic PALs refractory to conservative strategy has demonstrated that the EBV procedure is feasible, safe, and effective, some limitations should be highlighted. First in severely hypoxic patients the bronchoscopic interventional procedure may worsen respiratory failure. Secondly, in patients with bacterial super-infection valve positioning may facilitate post-obstructive pneumonia. Third, considering the risk of pneumothorax relapse, the timing of EBV removal should be carefully planned as data about the optimal management of these patients once air leaks resolution is achieved are lacking. However, in the most severe cases with an advanced stage of ARDS, at higher risk for iatrogenic PALs, the EBV procedure is likely to offer a reasonable minimally invasive therapeutic option.

## Data Availability

The datasets used and/or analyzed during the current study are available from the corresponding author on reasonable request.

## Abbreviations

PEEP: Positive end-expiratory pressure
EBV: Endobronchial valve
VILI: Ventilator-induced lung injury
MV: Mechanical ventilation
HFNC: High flow nasal cannula
NIV: Non-invasive ventilation
PAL: Persistent air leak
APF: Alveolar-pleural fistula
BPF: Bronco pleural fistula
RT-PCT: Real time polymerase chain reaction
SARS-CoV-2: Severe acute respiratory syndrome-coronavirus
COVID19: Coronavirus disease 19
ARDS: Acute respiratory distress syndrome
CRP: C reactive protein
CT: Computed tomography
ICU: Intensive Care Unit
